# ISU201 Enhances the Resolution of Airway Inflammation in a Mouse Model of an Acute Exacerbation of Asthma

**DOI:** 10.1155/2015/405629

**Published:** 2015-02-12

**Authors:** Yuka Hiroshima, Linda Garthwaite, Kenneth Hsu, Hyouna Yoo, Sang-Ho Park, Carolyn L. Geczy, Rakesh K. Kumar, Cristan Herbert

**Affiliations:** ^1^Inflammation and Infection Research, School of Medical Sciences, UNSW Australia, Sydney, NSW 2052, Australia; ^2^Isu Abxis Co., Ltd., 696-1 Sampyeong-dong, Bundang-ku, Seongnam 463-400, Republic of Korea

## Abstract

Glucocorticoids are commonly used for treating asthma and its exacerbations but have well-recognised adverse effects and are not always effective. Few alternative treatments exist. Using a murine model of an acute exacerbation of asthma, we assessed the ability of ISU201, a novel protein drug, to suppress the inflammatory response when administered after induction of an exacerbation. Sensitised mice were chronically challenged with a low mass concentration of aerosolised ovalbumin, and then received a single moderate-level challenge to simulate an allergen-induced exacerbation. ISU201 was administered to mice 2 and 8 hours later, while pulmonary inflammation and expression of mRNA for chemokines and proinflammatory cytokines were assessed after 4, 12, and 24 hours. Relative to vehicle-treated controls, ISU201 suppressed accumulation of pulmonary neutrophils and eosinophils, while accelerating the decline in CXCL1, TNF-*α*, and IL-6 in lavage fluid and lung tissue. ISU201 significantly reduced peak expression of mRNA for the chemokines *Cxcl9* and *Cxcl10*, the adhesion molecules *Icam1* and *Vcam1*, and the proinflammatory cytokines *Il1b*, *Il12p40*, and *Csf1*. The ability of ISU201 to promote resolution of inflammation suggests that it may have potential as an alternative to glucocorticoids in the management of asthma, including when administered after the onset of an acute exacerbation.

## 1. Introduction

Asthma is a common chronic inflammatory disorder, characterised by persistent inflammation of the airways with airway wall remodelling [1]. Acute exacerbations of asthma are episodes associated with severe airflow limitation that may require a change in treatment or a visit to an emergency department [2]. Exacerbations can be life-threatening and a substantial component of the health care costs of asthma is related to managing these events [3]. They are associated with increased expression of a variety of proinflammatory cytokines and enhanced airway inflammation which extends to the distal airways, with recruitment of large numbers of eosinophils and neutrophils [4].

Inhaled glucocorticoids are currently the mainstay of asthma therapy. These drugs are highly effective in reducing chronic inflammation and controlling clinical manifestations in the majority of patients [5], particularly when combined with long acting *β*2 agonists [6]. However, long-term use of inhaled steroids is associated with adverse effects, particularly when high doses are used [7, 8]. Furthermore, a proportion of patients have asthma which is relatively resistant to steroid treatment [9, 10], and these drugs are not always effective during acute exacerbations [11]. To date, there are very few alternatives to glucocorticoids [12].

ISU201, a novel protein drug developed by Isu Abxis Co., Ltd., may have potential for treating inflammatory diseases including asthma. The active moiety of ISU201 is the extracellular domain of the human cell-surface protein bone marrow stromal cell antigen 2 (BST2), stabilised for delivery by fusion with the Fc region of human IgG4 [13].

Our laboratory has developed a murine model of chronic asthma in which animals are sensitised to ovalbumin and chronically challenged with a low mass concentration of ovalbumin aerosol, to induce background lesions of chronic asthma including airway inflammation and airway wall remodelling [14]. We have also established a model of an allergen-induced acute exacerbation of asthma in which chronically challenged animals are subsequently exposed to an additional moderate-level challenge with allergen. This is associated with enhanced cytokine production and exaggerated inflammation of the airways and also involves smaller distal airways [15]. These models have been acknowledged to closely replicate key features of the disease in humans [16] and are useful for evaluating the effects of novel anti-inflammatory compounds. Using these models, we have recently demonstrated that long-term administration of ISU201 inhibits the progression of inflammation and airway wall remodelling, whereas administration prior to the induction of an exacerbation can limit the development of airway inflammation [17].

Resolution of inflammation is an actively regulated process [18] driven by a variety of endogenous lipid-derived mediators, including resolvins. We recently demonstrated the potential of resolvin E1 (RvE1) as a proresolution agent, by administering the drug to animals after the induction of an acute exacerbation [19]. Resolution-promoting compounds may have potential for the treatment of chronic inflammatory diseases and may be alternatives to steroids for the treatment of exacerbations of asthma. However, it is unknown whether ISU201 can limit the severity of an exacerbation when administered after it has been induced, a setting which more closely resembles that in which the drug would be used to treat patients with an exacerbation of asthma.

In this study, we investigated whether ISU201 could accelerate the resolution of airway inflammation when administered following induction of an allergen-induced acute exacerbation of mild chronic asthma. In parallel, we characterised its effects on the profile of expression of pro-inflammatory cytokines in the lung.

## 2. Materials and Methods

### 2.1. Animals and Treatments

All experimental procedures were approved by the UNSW Animal Care and Ethics Committee (ACEC 11/50A). Specific pathogen-free female BALB/c mice aged 7-8 weeks were obtained from Australian Bio-Resources (Sydney, Australia). Animals were housed on autoclaved bedding in individually ventilated cages (VentiRack) with a 12-hour light/dark cycle and food and water* ad libitum*.

**Figure 1:**
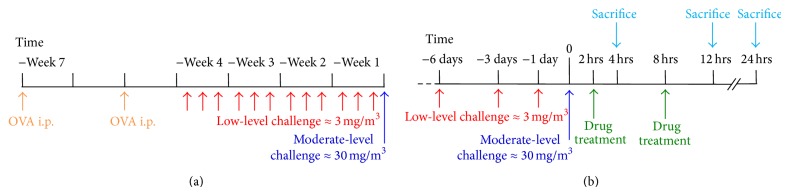
Timeline showing (a) sensitisation/challenge in the model of an acute exacerbation of chronic asthma and (b) timing of drug administration and sample collection following induction of an acute exacerbation.

Sensitisation and inhalational challenge with ovalbumin aerosol were performed as previously described [15]. In brief, animals were sensitised by intraperitoneal injection of 50 *μ*g of ovalbumin (Grade V; Sigma, Australia) adsorbed to 1 mg of Alum in saline, 21 and 7 days prior to commencement of inhalational challenge. Mice were challenged via the airways with a low mass concentration of an ovalbumin aerosol (3 mg/m^3^) in a whole body inhalation exposure chamber (Unifab Corporation, Kalamazoo, MI) for 30 minutes/day on 3 days/week for 4 weeks to establish background lesions of chronic asthma. The concentration of ovalbumin within the chamber was continuously monitored using a DustTrack 8250 instrument (TSI, St Paul, MN). An acute exacerbation was induced by a subsequent single 30-minute challenge with a higher concentration (30 mg/m^3^) of aerosolised ovalbumin, which causes enhanced airway inflammation extending to the distal airways. The treatment protocols are outlined in Figure 1(a).

ISU201 was prepared and supplied by Isu Abxis Co., Ltd., (Seoul, Korea). Experimental groups of 6 mice were treated with ISU201 in saline (20 mg/kg; i.p.) or with water-soluble dexamethasone (1 mg/kg; p.o.) at 2 and 8 hours after an acute exacerbation. These were compared to mice which received vehicle alone (saline; i.p.) after the exacerbation. Samples were collected at 4, 12, or 24 hours after the exacerbation (Figure 1(b)). Naïve control mice were also assessed in parallel.

### 2.2. Assessment of Inflammatory Response

Mice were killed by exsanguination following an overdose of sodium pentobarbital and the lungs perfused with saline to removed blood from the pulmonary capillary bed. Bronchoalveolar lavage (BAL) was performed by cannulating the trachea and washing the lungs with 2 × 1 mL of ice-cold PBS. BAL fluid was collected for measurement of cytokine concentrations and a differential count of cells was performed on 300 cells in Leishman-stained cytospin preparations.

The single-lobed left lung was inflated with OCT and frozen. Sections (5 *μ*m) were cut, stained with haematoxylin and eosin, and assessed for peribronchiolar inflammation.

Tissue from the middle lobe of the right lung was collected in TriReagent (Sigma) for isolation of total RNA. The upper lobe of the right lung was collected for assessment of eosinophil accumulation by assessing eosinophil peroxidase as previously described [20]. The lower lobe of the right lung was lysed in protein lysis buffer (Cell Signalling Technology, Beverly, MA) and protein concentration in the lysate was determined using a protein assay (Bio-Rad, Gladesville, New South Wales, Australia). Cytokines in tissue lysate were quantified by ELISA using equal amounts of total protein.

### 2.3. Enzyme Immunoassays

Concentrations of proinflammatory cytokines IL-6 and TNF-*α* and chemokines CXCL1 and CCL11 were measured in BAL fluid and lung tissue lysates using ELISA kits (R&D Systems) according to the manufacturer's recommendations.

### 2.4. Quantitative Real-Time PCR

Total RNA (5 *μ*g) from lung tissue was treated with DNase (Turbo DNase; Ambion, Soresby, Australia) and reverse-transcribed using Superscript III (Life Technologies). Quantitative real-time PCR was performed on a Light Cycler 480 (Roche) to assess expression of mRNA for a custom panel of 95 genes including cytokines, chemokines, adhesion molecules, growth factors, and housekeeping genes. Amplified products were detected using SYBR green (Bioline) and expression was normalised to* Hprt*.

### 2.5. Statistical Analysis

Data are presented as mean ± SEM. A one-way Analysis of variance (ANOVA) followed by a Holm-Sidak multiple comparison test was used to identify differences between experimental groups. A *P* value of <0.05 was considered significant. GraphPad Prism version 6.04 (GraphPad Software, San Diego, CA) was used for the analysis of data and preparation of graphs.

## 3. Results

No adverse events occurred as a result of sensitisation, inhalational challenge, or drug treatment.

### 3.1. ISU201 Promotes Resolution of Inflammation

We investigated the effect of treatment with ISU201, after inducing an acute exacerbation of experimental asthma, on pulmonary inflammation and cytokine production.

**Figure 2:**
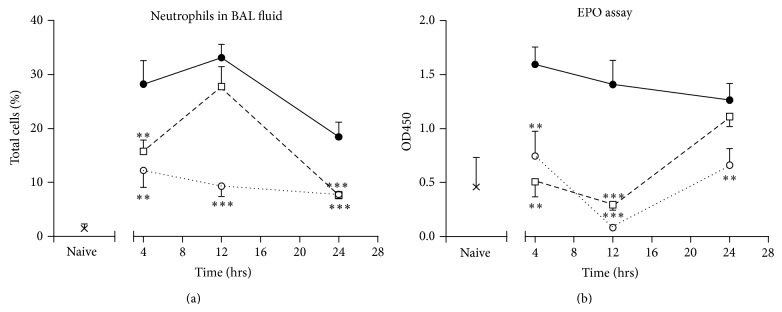
Effects of drug treatment when administered after an exacerbation of experimental asthma on (a) neutrophils in lavage fluid (b) eosinophils in lung tissue, assessed by colorimetric assay for EPO. Animals were treated with vehicle (—●—), ISU201 (- -□- -), or dexamethasone (⋯○⋯) at 2 and 8 hours after induction of an exacerbation. Data are mean ± SEM (*n* = 6 animals per group). Significant differences relative to vehicle-treated animals at each time point are shown as (^**^ *P* < 0.01) and (^***^ *P* < 0.001).

Following an allergen-induced exacerbation, the percentage of neutrophils in BAL fluid was increased at 4 hours (19.3-fold ±3.0 relative to naive mice), was maximal after 12 hours (22.7-fold ±1.7 relative to naive mice), and had declined at 24 hours (9.5-fold ±0.8 relative to naive mice). In mice treated with ISU201, the percentage of neutrophils in lavage fluid was significantly reduced relative to vehicle-treated animals after both 4 and 24 hours (Figure 2(a)) although numbers recruited at 12 hours were elevated. This contrasted markedly with dexamethasone, which significantly reduced neutrophil recruitment at all time points. Eosinophil recruitment to the lung, measured as eosinophil peroxidase, was greatest after 4 hours (3.5-fold ±0.4 relative to naive mice) and slowly declined after 12 and 24 hours. ISU201 significantly reduced the number of eosinophils in lung tissue at 4 and 12 hours after an acute exacerbation relative to vehicle-treated animals (Figure 2(b)). In this case, the response was similar to suppression seen with dexamethasone at all time points tested.

### 3.2. ISU201 Suppresses Production of Proinflammatory Cytokines in the Lung

**Figure 3:**
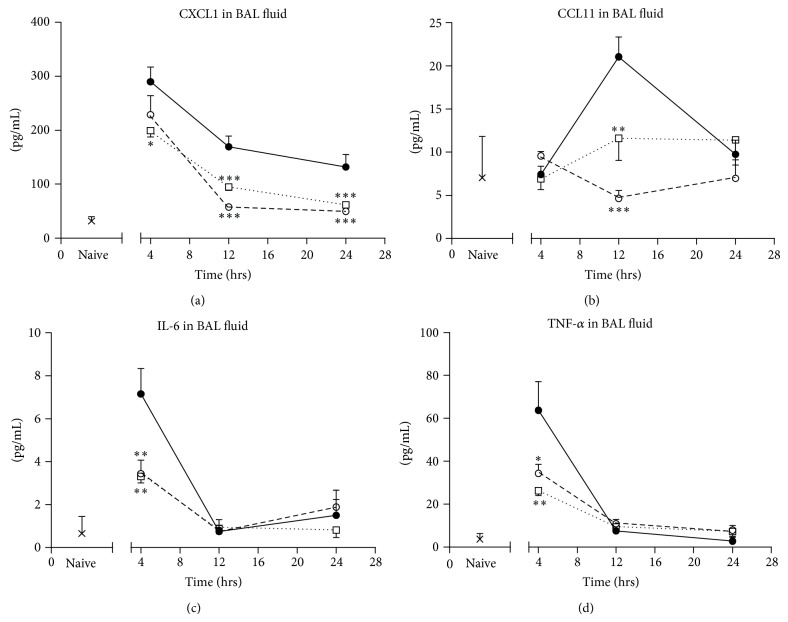
Effects of drug treatment on production of proinflammatory cytokines (a) CXCL1, (b) CCL11, (c) IL-6, and (d) TNF-*α* in BAL fluid. Animals were treated with vehicle (—●—), ISU201 (- -□- -), or dexamethasone (⋯○⋯) at 2 and 8 hours after induction of an exacerbation. Data are mean ± SEM (*n* = 6 animals per group). Significant differences relative to vehicle-treated animals at each time point are shown as (^*^ *P* < 0.05), (^**^ *P* < 0.01), and (^***^ *P* < 0.001).

The increases in cytokine concentrations seen in lavage fluid in vehicle-treated animals were markedly suppressed in animals treated with ISU201 after an acute exacerbation. ISU201 reduced CXCL1 levels in BAL fluid by 44% at 12 hours and by 53% at 24 hours compared to vehicle-treated controls (Figure 3(a)). CCL11 levels were also significantly reduced by ISU201 (45% reduction compared to vehicle-treated animals) in BAL fluid collected at 12 hours (Figure 3(b)). Concentrations of IL-6 and TNF-*α* in BAL fluid were significantly reduced at 4 hours in animals treated with ISU201 (Figures 3(c)-3(d)). The suppression of CXCL1, IL-6, and TNF-*α* by ISU201 was equivalent to that achieved with the glucocorticoid dexamethasone.

**Figure 4:**
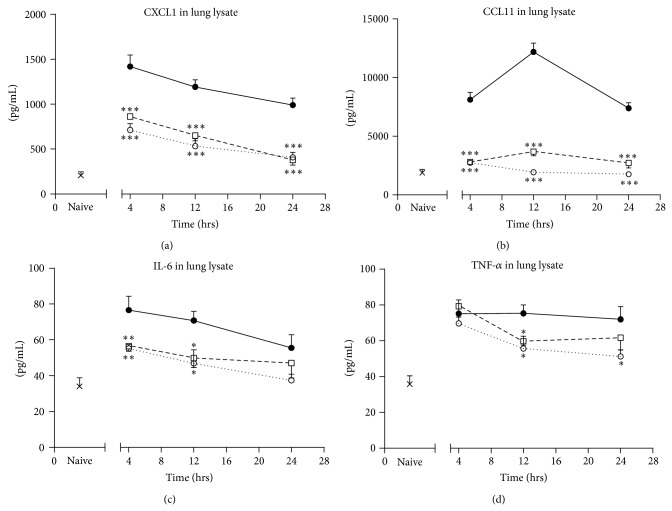
Effects of drug treatment on production of proinflammatory cytokines (a) CXCL1, (b) CCL11, (c) IL-6, and (d) TNF-*α* in lung tissue lysate. Animals were treated with vehicle (—●—), ISU201 (- -□- -), or dexamethasone (⋯○⋯) at 2 and 8 hours after induction of an exacerbation. Data are mean ± SEM (*n* = 6 animals per group). Significant differences relative to vehicle-treated animals at each time point are shown as (^*^ *P* < 0.05), (^**^ *P* < 0.01), and (^***^ *P* < 0.001).

In vehicle-treated mice, concentrations of proinflammatory cytokines in lung tissue lysates were significantly increased 4 hours after exacerbation and remained elevated for up to 24 hours relative to naïve animals. Levels of the chemokines CXCL1 and CCL11 were 4.9-fold and >1000-fold higher in lysate than in BAL fluid. Treatment with ISU201 after an acute exacerbation significantly reduced both CXCL1 and CCL11 relative to vehicle-treated animals at all times assessed (Figures 4(a)-4(b)). ISU201 also reduced the concentration of IL-6 at 4 and 12 hours, while the concentration of TNF-*α* was only reduced at 12 hours (Figure 4). ISU201 suppressed CXCL1, CCL11, and IL-6 in lung lysate as effectively as the control drug dexamethasone. However, dexamethasone suppressed TNF-*α* at both 12 and 24 hours.

### 3.3. Effect of Treatment on Expression of mRNA for Proinflammatory Cytokines

**Figure 5:**
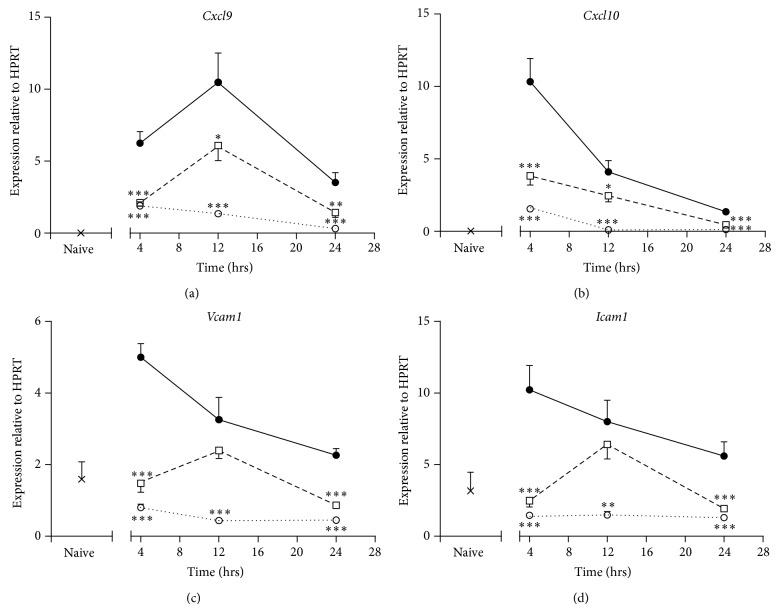
Effects of treatment with ISU201 on expression of mRNA for chemokines (a)* Cxcl9,* (b)* Cxcl10* and adhesion molecules (c),* Vcam1,* and (d)* Icam1*. Animals were treated with vehicle (—●—), ISU201 (- -□- -), or dexamethasone (⋯○⋯) at 2 and 8 hours after induction of an exacerbation. Data are mean ± SEM (*n* = 6 animals per group). Significant differences relative to vehicle-treated animals at each time point are shown as (^*^ *P* < 0.05), (^**^ *P* < 0.01), and (^***^ *P* < 0.001).

We next examined the effect of treatment with ISU201, after the induction of an exacerbation of asthma, on profiles of mRNA expression of a panel of key proinflammatory mediators. In vehicle-treated mice, the expression of mRNA for* Cxcl9* and* Cxcl10* was elevated 343-fold and 295-fold, respectively, 4 hours after an exacerbation, relative to naïve mice. Figures 5(a) and 5(b) show that ISU201 was moderately effective in suppressing mRNA levels of these chemokines, relative to vehicle-treated animals, at all times assessed, while dexamethasone causes marked suppression. Adhesion of leukocytes to the vascular endothelium, which is critical for their extravasation, is modulated by induction of ICAM-1 and VCAM. Genes encoding* Vcam1* and* Icam1* were elevated 3.1-fold and 3.2-fold, respectively, in lung tissue from vehicle-treated mice 4 hours after an exacerbation. In animals treated with ISU201, the increase in* Vcam1* and* Icam1* mRNA was completely suppressed 4 and 24 hours after an exacerbation (Figures 5(c)-5(d)).

**Figure 6:**
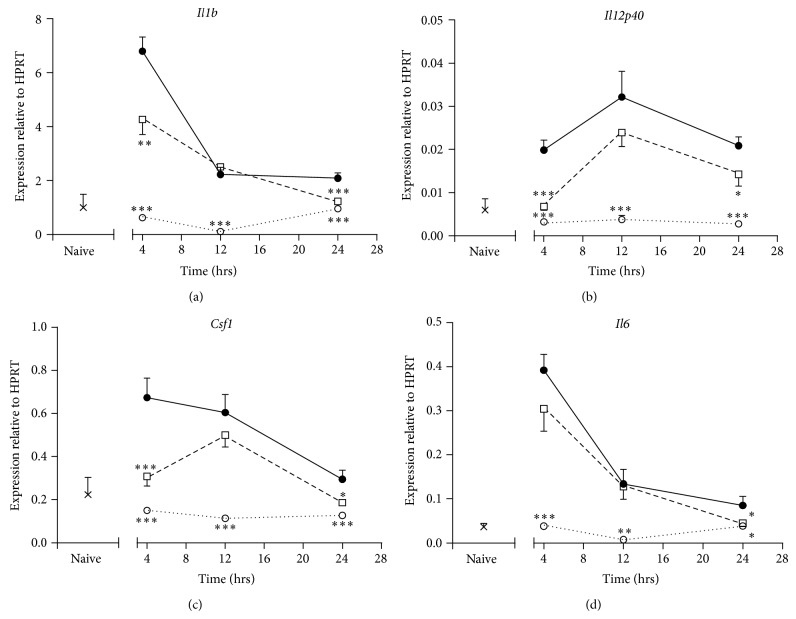
Effects of treatment with ISU201 on expression of mRNA for proinflammatory cytokines (a)* Il1b,* (b)* Il12p40,* (c)* Csf1,* and (d)* Il6*. Animals were treated with vehicle (—●—), ISU201 (- -□- -), or dexamethasone (⋯○⋯) at 2 and 8 hours after induction of an exacerbation. Data are mean ± SEM (*n* = 6 animals per group). Significant differences relative to vehicle-treated animals at each time point are shown as (^*^ *P* < 0.05), (^**^ *P* < 0.01), and (^***^ *P* < 0.001).

ISU201 also significantly reduced expression of mRNA for the proinflammatory cytokines* Il1b*,* Il12p40,* and* Csf1* in lungs collected at 4 and 24 hours (Figures 6(a)–6(c)). Expression of mRNA for* Il6* was significantly reduced by ISU201 only at 24 hours (Figure 6(d)). Expression of mRNA for these mediators was significantly reduced in mice treated with dexamethasone at all time points assessed, to the level seen in naïve mice.

Treatment with ISU201 did not significantly reduce expression of mRNAs for the chemokines* Ccl2*,* Ccl3*,* Ccl11*, and* Cxcl1* or for* Il13*, while* Ccl4* was reduced only at 24 hours. In contrast, expression of these mRNA species was significantly reduced in lungs from animals treated with dexamethasone at every time point examined (see Supplementary Figure  1 in Supplementary Material available online at http://dx.doi.org/10.1155/2015/405629).

## 4. Discussion

Preclinical investigation of novel therapeutic agents requires not only appropriate animal models but also clinically relevant approaches to administration of drugs. In this study, we used our well-validated murine model to investigate the effects of ISU201 on inflammation and cytokine production in an allergen-induced experimental acute exacerbation of chronic asthma. Importantly, we assessed the response to treatment when the drug was administered after the induction of the exacerbation, to more closely simulate the clinical setting in which the drug might be used.

We showed that treatment with ISU201 reduced the accumulation of neutrophils in BAL fluid collected at 4 and 24 hours and of eosinophils in lung tissue collected at 4 and 12 hours. Assessment of levels of the chemokines CXCL-1 (GRO-*α*, a neutrophil chemoattractant) and CCL11 (eotaxin-1, an eosinophil chemoattractant) confirmed that these were elevated following exacerbation. In keeping with its negative effect on neutrophil and eosinophil recruitment and in a manner similar to that seen with dexamethasone, ISU201 markedly suppressed production of these chemokines. Suppression was obvious in BAL fluid and particularly in lung lysates, in which levels of chemokines were high and remained elevated for 24 hours or longer. Importantly, the effects of ISU201 on cytokine expression were apparent at multiple time points and were frequently of a similar magnitude to those of dexamethasone, which in this study was used as a positive control, at a much higher dose (relative to body weight) than what would be employed in patients [21].

We also showed that when given after induction of an exacerbation, ISU201 potently suppressed induction of chemokine genes important in lymphocyte recruitment.* Cxcl9* (known as monokine induced by interferon-*γ* (MIG)) and* Cxcl10* (known as interferon-induced protein 10 (IP-10)) were significantly reduced in lung tissue at all time points assessed, while the expression of mRNA for* Il1b*,* Il12p40* and* Csf1* was reduced at 2 of the 3 time points. For several other chemokines, levels of mRNA were increased following induction of an exacerbation (Supplementary Figure  1) but were not significantly altered by ISU201, whereas they were reduced by dexamethasone, indicating diverging pathways of suppression. Unsurprisingly, the profile and time course of changes in expression of cytokine mRNA did not correlate perfectly with protein levels detected in BAL fluid or lysates. This may relate to the secretion of stored protein or the assessment of airway* versus* peripheral cellular sources of these mediators.

There is a significant need for alternatives to steroids for treatment of exacerbations and for management of chronic asthma when steroids are not effective. Collectively, our data suggest that ISU201 has a potent and broad spectrum of activity, which affects a variety of cells and mediators. Thus it seems reasonable to suggest that ISU201 may be useful as an alternative to steroids for exacerbations. In this context, it is noteworthy that a number of recently developed treatments which specifically target individual cytokines, including IL-4R*α*, IL-5, and TNF-*α*, have been ineffective in clinical trials or at best useful in selected populations of patients [22–24]. This may in part be due to cytokine redundancy, in which cytokines with similar functions are not targeted. We believe that novel agents which target multiple cytokines and chemokines, such as ISU201, are more likely to be clinically effective than therapies which target a single molecule or pathway [25].

The present study confirms and extends our previous work demonstrating that ISU201 has potent anti-inflammatory activity [17]. We have shown that administration of ISU201 after induction of an exacerbation limits the peak accumulation of neutrophils and eosinophils, as well as the expression of mRNA for proinflammatory cytokines. Treatment with ISU201 also promotes or accelerates the decline in numbers of inflammatory cells and levels of expression of cytokine mRNA. Thus it appears to accelerate the process of resolution of inflammation. The design of our study is in contrast to preclinical evaluations of the potential of novel anti-inflammatory compounds in which the drugs are only given prior to the induction of inflammation. Relatively few studies examine the effect of treatment in established inflammation, which is a more clinically relevant setting [19, 26, 27].

We recognise that the distinction between anti-inflammatory and proresolution activity is blurred. Typically, anti-inflammatory agents reduce leucocyte recruitment and inhibit cytokine and chemokine production, whereas resolution-promoting agents enhance apoptosis of leucocytes and stimulate phagocytosis [28]. However, some drugs modulate both aspects of the inflammatory process. Notably, glucocorticoids are potent anti-inflammatory agents but can also promote phagocytosis [29] and apoptosis [30, 31]. In contrast, RvE1 promotes the resolution of inflammation when administered to animals after the induction of an exacerbation but has limited anti-inflammatory activity when administered prior to an exacerbation of asthma [19]. An ideal drug to treat inflammatory disease would suppress the initiation of inflammation and also enhance resolution [18]. Our previously published data indicate that ISU201 functions as an anti-inflammatory agent [17], while the results of the present study establish that it is also a proresolution agent.

Currently, the mechanism(s) of action of ISU201 are not completely understood. We recently demonstrated that ISU201 suppresses cytokine production by alveolar macrophages and lymphocytes, as well as reducing histone H4 acetylation in airway epithelial cells [17]. The active moiety of ISU201 (BST2) has been shown to decrease the adhesion of monocytes to human umbilical vein endothelial cells [13] so it is also possible that ISU201 may inhibit/impede the interaction between leucocytes and adhesion molecules on endothelial cells during inflammation. Results from the current study have also demonstrated that treatment of animals with ISU201 reduces the expression of mRNA for* Vcam1* and* Icam1,* which may contribute to inhibiting the adhesion and emigration of inflammatory cells into the lungs. Further investigation of the mechanism(s) of action of ISU201 is the focus of ongoing work in our laboratories.

## 5. Conclusions

We have previously demonstrated that the novel protein drug ISU201 can prevent the progression of airway inflammation and remodelling in a murine model of chronic asthma and that pretreatment can suppress the acute inflammatory response associated with an allergen-induced exacerbation. In the current study, we have demonstrated that when administered to animals after the induction of an acute exacerbation of asthma, ISU201 effectively reduces the magnitude of the inflammatory response and accelerates the resolution of inflammation. ISU201 also suppresses the increase in expression of mRNA for a broad range of inflammatory cytokines. Thus, this drug may have potential as an alternative to glucocorticoids in the management of asthma and its acute exacerbations.

## Supplementary Material

Treatment with ISU201 after the induction of an acute exacerbation had no effect on the expression of mRNA for the chemokines Ccl2, Ccl3, Ccl11 and Cxcl1 or for Il13, and reduced mRNA for Ccl4 only at 24 hours. In contrast, treatment with dexamethasone reduced expression of these mRNA species at every time point examined.
