# Supplementary figures and images for: ISU201 Enhances the Resolution of Airway Inflammation in a Mouse Model of an Acute Exacerbation of Asthma
Source: Mediators Inflamm. 2015 Feb 12;2015:405629. doi: 10.1155/2015/405629 (PMC4342076; doi:10.1155/2015/405629)

Manuscript 405629v2  
Supplementary Figure 1

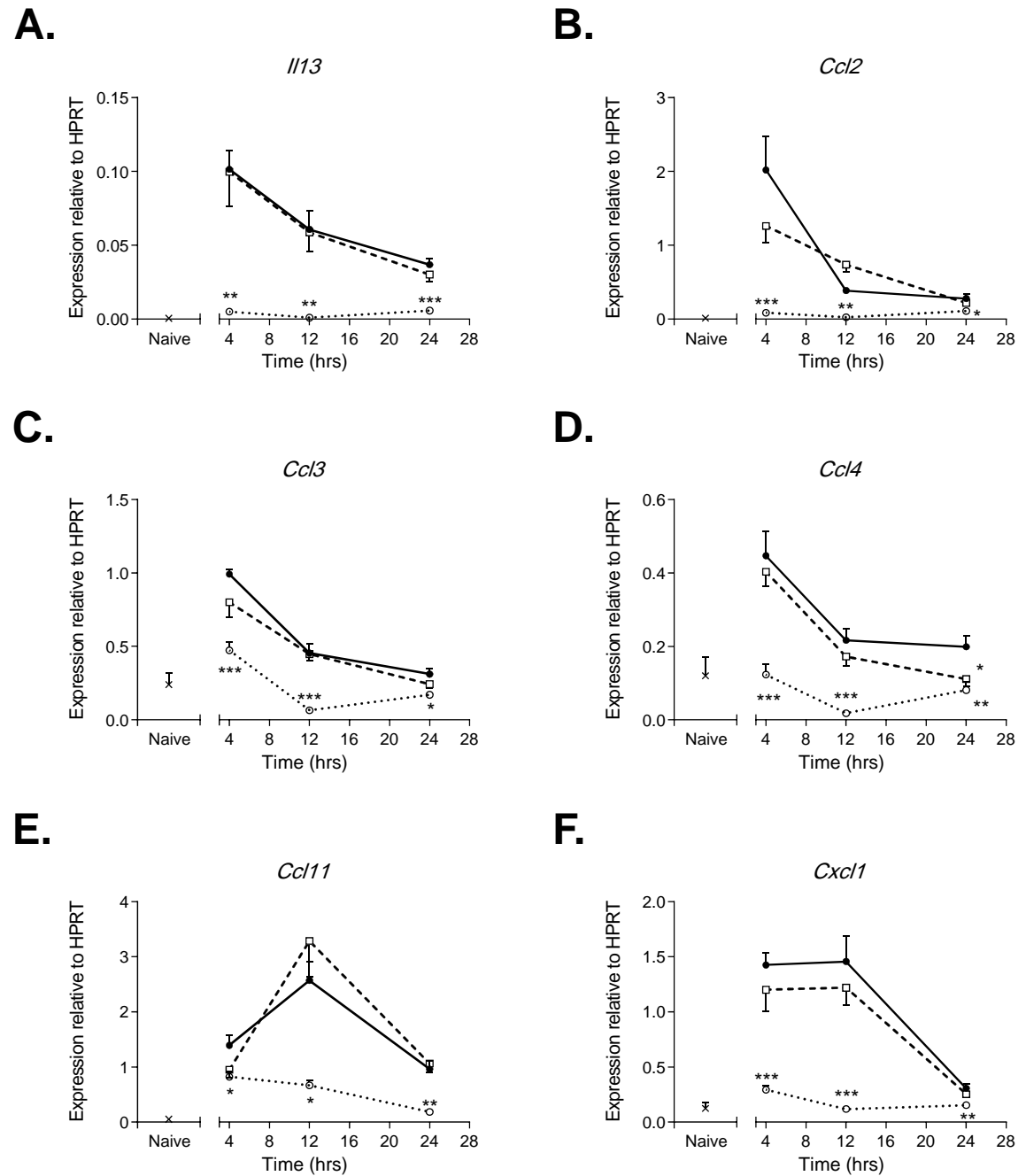

Supplement: Supplementary file 1 — Treatment with ISU201 after the induction of an acute exacerbation had no effect on the expression of mRNA for the chemokines Ccl2, Ccl3, Ccl11 and Cxcl1 or for Il13, and reduced mRNA for Ccl4 only at 24 hours. In contrast, treatment with dexamethasone reduced expression of these mRNA species at every time point examined. [file 405629.f1.pdf]
